# Supplementary material for: SyNDI: synchronous network data integration framework
Source: BMC Bioinformatics. 2018 Nov 6;19:403. doi: 10.1186/s12859-018-2426-5 (PMC6219086; doi:10.1186/s12859-018-2426-5)
Supplement: Supplementary file 5 — Files of the example presented in Additional file 4. This additional file is a zip package that contains all files used in Additional file 4. Detailed descriptions of each of these files are presented in a README file included in this zip package. (GZ 30411 kb) [file 12859_2018_2426_MOESM5_ESM.gz › SyNDITools/Meme2Fimo/meme_4.9.0_4.tar.gz!/meme_4.9.0/website/cgi-bin/centrimo.tmpl]

CentriMo - Submission form


Central mode tests regions centered on the sequence for enrichment. This
is useful for ChIP-seq data as you expect your ChIPed TFBS to appear in
areas of maximum tag density. If you prepare sequences centered on these
positions of maximum confidence then CentriMo can help confirm that your
expected motif does correspond to the ChIPed TF.

Local mode tests all possible regions for enrichment.
The downside is this mode must do many more tests than central mode which
makes it slower and also less sensitive in the central regions due to the
larger multiple test correction.

[ close ]

The binomial test compares the number of sequences that had their best
matches in the region to the expected number of sequences under the assumption
that matches would be randomly distributed.

Adding a second comparative dataset allows use of the Fisher exact test
(FET) to sort the regions found by the binomial test. The FET compares the
number of sequences that have their best matches in the region to the
number found in the comparative set.

[ close ]

Select a file of FASTA formatted
DNA sequences or paste in actual FASTA formatted DNA sequences to search for enriched regions.

See the sample sequences
to get an idea of input that works well for CentriMo.

[ close ]

Select a file of FASTA formatted
DNA sequences or paste in actual FASTA formatted DNA sequences to compare to the other sequence set.

See the sample sequences
to get an idea of input that works well for CentriMo.

[ close ]

CentriMo can use databases installed on the server or search a user
uploaded database in MEME DNA motif
format by selecting the "Upload Your Own Database" option. For more
information browse the
supported databases.

[ close ]

An uploaded database is a normal MEME DNA motif file with lots of motifs. The uploaded database must
be smaller than 5MB.

[ close ]

The email is used to notify you of your results.

[ close ]

The job description will be included in the notification email you
receive and in the CentriMo output.

[ close ]

By default CentriMo will determine the background Markov model from
the provided sequences. Alternately a file containing a background model may be input here.

The downloadable version of the MEME Suite contains a script named
"fasta-get-markov" that you can use to create background model files in
the correct format from FASTA sequence files.

[ close ]

Choose between single and double strand scanning. The flip RC position
option causes any matches on the reverse strand to have their position
mirrored around the center of the sequence. This option was enabled by
default in the original released version of CentriMo.

[ close ]

Specify a minimum score for a match to be considered. If a sequence does
not have any matches which meet this minimum score for a given motif, then
that sequence will not be considered for that motif.

If the "optimize score" option is selected then all distinct scores for
matches that did better than the minimum score will be tested as alternative
minimum score thresholds. Enabling this option will slow down the program
and increase the multiple testing correction.

[ close ]

This option limits the maximum region size that CentriMo will test.
This option is useful if you have sequences longer than the normal 500bp
and can help cut-down the multiple testing correction. When this option is
not supplied CentriMo will test region sizes up to one less than the maximum
number of places that a given motif can align to the sequence.

[ close ]

This is the E-value threshold for reporting enriched regions for motifs.
If multiple enriched regions overlap then the region with the best p-value
and smallest size will be output.

[ close ]

This option enables you to store the sequence identifies which have
their best match in the best region for each motif. This option can
potentially make the CentriMo output file size much larger.

[ close ]

|  |  |
| --- | --- |
|  | MEME Suite  The MEME Suite web application requires the use of JavaScript  Javascript doesn't seem to be available on your browser.  Version  Use this form to determine centrally/locally enriched motifs in a set of sequences of the same length. To use central enrichment mode, ideally the sequences should each be 500-bp long, and centered on the position of "maximum confidence" (e.g., maximum tag density) in a ChIP-seq peak.  Data Submission Form Determine central or localized enrichment of motifs.  Select where to search for enrichment  Centrally  Any Localization   Select how enrichment is determined  Binomial Test  Binomial Test + Fisher Exact Test (comparative)   Input the sequences  Enter DNA sequences in which you want to analyse motif locality  or paste the sequences   Input the comparative sequences  Enter DNA sequences for comparing motif locality  or paste the sequences   Input the motifs  Use Submitted Motifs  Upload Your Own Database  Input motifs to upload  Select a MEME motif database file.   Input job queue details  Enter your email address.  Re-enter your email address.     Optionally enter a job description.   ▶ ▼  Advanced options hidden modifications! [Reset]  Scan both DNA strands?  scan both  scan both + flip RC position  scan given only  Set a minimum acceptable match score (bits) score ≥   optimize score?  Set the maximum allowed width of region  region width ≤   Set *E*-value threshold for reporting enriched regions *E*-value ≤   Include sequence IDs  Include a list of matching sequence ids  Use a custom background? Custom background: |
